# Examining associations between oral cancer mortality and economic development of 13 European countries: an ecological and correlational study

**DOI:** 10.1186/s12903-024-05134-4

**Published:** 2025-01-07

**Authors:** Klára Boruzs, Eszter Barbara Bán, Viktor Dombrádi, Gábor Bányai, Gergő József Szőllősi, Klára Bíró

**Affiliations:** 1 Institute of Health Economics and Management, Faculty of Economicsand Business, University of Debrecen, Debrecen, Hungary; 2 Oro-Maxillofacial Surgery and Stomatology Clinic, Health Care Service Units, Clinics University of Debrecen Clinical Center, University of Debrecen, Debrecen, Hungary; 3 Coordination Center for Research in Social Sciences, Faculty of Economics and Business, University of Debrecen, Debrecen, Hungary

**Keywords:** Oral health, Oral cancer, GDP, Economic development, Oral cancer prevalence

## Abstract

**Background:**

Cancer is a significant public health issue all over the world. The diagnosis, treatment and follow-up of cancer patients are a huge health, economic and social burden for societies. The link between the state of health of a country and its economic performance has been proven by numerous studies. The aim of this study was to examine the age-standardized death rate of oral cancer in relation to the economic development of 13 European countries.

**Methods:**

Aggregated data were collected from the European Health for All (HFA-DB) database until 2019 and analysed using second degree polynomial functions and correlation analyses, followed by time-series analysis involving vector autoregressive models.

**Results:**

We found that in 10 of the 13 surveyed countries, the age-standardized death rate of oral cancer initially increased with GDP growth and then showed a downward trend above a certain level of economic development. Austria had a weak but significant positive effect with the second lag of GDP, Hungary had a significant negative effect with the first lag of GDP, and Italy had a significant negative effect with the second lag of GDP. In most cases, both the first and second lags of GDP changes were not statistically significant, indicating that short-term fluctuations in GDP do not directly influence changes in oral cancer mortality rates. Overall, while there are clear long-term associations between GDP and oral cancer mortality rates, the immediate causal effects of GDP changes on mortality rates are limited, suggesting that other factors and longer-term dynamics could play a more crucial role in this relationship.

**Conclusions:**

An increase in a country’s economic development alone does not guarantee a decrease in the number of oral cancer patients. Therefore, in order to reduce the number of cases of oral cancer, strengthening education and prevention are essential.

## Background

According to GLOBOCAN statistics, the number of new cancer cases is estimated to reach 19.3 million in 2020, with an estimated 10 million cancer deaths globally. Although Europe’s population represents only 9.7% of the total population, it makes up 22.8% of the cancer population and 19.6% of cancer deaths [1].

The classic term oral cavity tumours refer to tumours of the oral cavity (front two-thirds of the tongue, gums, floor of the mouth, hard palate and mucous membrane of the cheek) and the palate (back one-third of the tongue, root of the tongue, soft palate, tonsillar fossae). Some listings also include tumours of the lip in the group of oral cavity tumours [2].

Oral cancers were the 18th most common cancer in 2020, with oral cavity cancers being the 26th most common. In that year, 377,713 new cases of oral cancer and 98,412 cases of oral cavity cancer were registered worldwide. In 2020, oral cancers caused 177,757 deaths and oropharyngeal cancers caused 48,143 deaths globally [1].

Current research suggests that by 2030, there will be an increase of 22 million in cancer incidence and 13 million in mortality worldwide [3]. This is due to the continuing growth and ageing of the world population and, in many cases, changes in the prevalence and distribution of risk factors linked to socio-economic development [4].

Although the incidence of oral cancer is decreasing in developed countries, it has increased in many European countries. Research suggests that this may be due to differences in social habits, prevention, education and the quality of medical records [5].

The diagnosis, treatment and follow-up of cancer patients are a major health, economic and social burden for societies. The economic losses due to early morbidity and mortality have to be taken into account: in addition to the direct health care, prevention and care costs, the fact that some patients are unable to return to work is also a problem [6].

Smoking, alcohol consumption and human papillomavirus (HPV) infection could be considered as the most important aetiological factors in the development of oral cancer [7]. However, other non-health related factors may also increase the onset of oral cancer.

GDP is the most important indicator of economic activity, but it does not provide an accurate measure of the material well-being of a country’s population, for which alternative indicators may be more appropriate [8]. Although GDP does not give a comprehensive picture of either general or economic well-being, it provides a vast amount of information closely related to well-being and remains one of the most important indicators in economic statistics.

According to the Bureau of Economic Analysis, there are two approaches to calculating GDP. The expenditure approach measures GDP as the sum of consumption, investment, government spending and net exports. On the income side, GDP is measured as the sum of income from production [9]. The third way of calculating GDP is the sum of value added.

According to the Hungarian Central Bureau for Statistics definition, “Gross National Income (GNI) is an indicator derived from GDP, and takes into account primary income received from abroad and paid to abroad” [10].

The World Bank classifies countries’ economies into four income groups based on their per capita national income (GNI): low (per capita GNI of US$ 1,085 or less), lower middle (per capita GNI between US$ 1,086 and US$ 4,255), upper middle (per capita GNI between US$ 4,256 and US$ 13,205) and high (per capita GNI of US$ 13,205 or more). All the countries in our study are high income countries [11].

The relationship between health and economic performance has been the subject of numerous studies. According to several studies, higher educational attainment, higher life expectancy, declining population fertility, lower government consumption, better maintenance of the rule of law, lower inflation and improved external trade conditions increase the rate of economic growth [12–14]. Human capital is therefore an important component in the growth of economic performance. The most noteworthy determinants of human capital are education and health, the latter now being considered more important [13, 15].

The positive impact of economic well-being on human capital is well known. Higher incomes provide better access to goods and services, while a nutritious diet, safe drinking water, better hygienic conditions and higher quality health care improve the health status of the population. There are also a number of studies on the impact of human capital on economic performance, showing that health has a significant positive impact on economic growth [14, 16, 17].

This is due to its direct impact on production capacity. Healthy workers have greater physical and mental performance, so they produce more and earn higher wages, take less sick leave because of their own or their relative’s health problems. Better health also has an impact on the economy through education. Healthy children perform better and have less sick leave in school. Reductions in morbidity and mortality increase investment in education. Increased life expectancy also leads to increased savings for retirement [17].

Taking all the aforementioned information into account, the aim of this study was to investigate the age-standardized death rate of oral cancer in the context of economic development in European countries. In an earlier research meaningful conclusion could be derived when examining the relationship between economic growth and the prevalence of mental disorders in Poland, Czech Republic, Slovakia and Hungary [18]; thus, the goal was to use a similar approach to gain better insight into this topic. Also, future studies can use the findings to identify the underlying causes and to follow and monitor the trend of the correlation in order to adjust the effectiveness of prevention activities accordingly.

## Methods

### Data collection

Using minimum 27 and maximum around 50 years of data a study on ecological correlations was executed. After calculating the coefficients of determination, our analysis selected those European countries where R2 regarding GDP and mortality was equal or greater than 0.7, which implies a strong correlation [19, 20]. Countries with significant gaps or several incomplete data were excluded from the analysis to ensure the robustness and reliability of our results. However, exclusion of these countries may introduce bias, as it limits the generalizability of the findings to countries with less complete data, since countries with incomplete data may had different economic and health dynamics compared to those included. As a consequence, the following 13 countries were participated in the analysis: Belgium, Germany, Spain, Croatia, The Netherlands, Poland, Romania, Austria, France, Hungary, Portugal, Denmark and Italy.

The data and results presented in this paper follow an ecological and correlational study design. Data on lip/oral cavity/pharynx malignant cancer mortality were derived from the World Health Organization (WHO) European Health for All database (HFA-DB) online database [21], where information on health status and health resource utilization is available. The standardised indicator which we used shows the mortality rate from malignant neoplasms of the lip, oral cavity and pharynx per 100,000 persons. Data on GDP per capita are taken from the Penn World Table, an online database [22] containing information on the income of 183 countries between 1950 and 2019.

### Analysis

The trends in the age-standardized death rate of oral cancer in relation to GDP was initially analysed by fitting second-degree curves to the examined factors in an ecological design. However, limitations could be inherent in ecological analyses, including ecological fallacy, where associations observed at the population level may not necessarily hold at the individual level. Subsequently, Spearman correlation coefficients were calculated with their matched p-values to investigate the relationship between GDP and oral cancer mortality across different countries. Since most data was initially non-stationary, as confirmed by the Augmented Dickey-Fuller test, differencing was performed to achieve stationarity. Following this process and the preliminary analysis, vector autoregressive (VAR) models were employed to further examine the dynamic interactions and potential causality between GDP and oral cancer mortality over time providing insights into the temporal dependencies and the directional influences between the variables analysed. This allowed for the modelling of dynamic relationships between variables, accounting for lagged effects and influences over time, therefore it was considered as suitable for capturing the delayed impact of economic changes on health outcomes, such as the effect of GDP on oral cancer mortality. Statistical analyses were conducted using Stata statistical software (version 13.0, StataCorp, College Station, TX, USA), with a p-value of less than 0.05 considered indicative of statistical significance.

## Results

### Country-specific descriptive analysis between oral cancer mortality and economic development

**Fig. 1:**
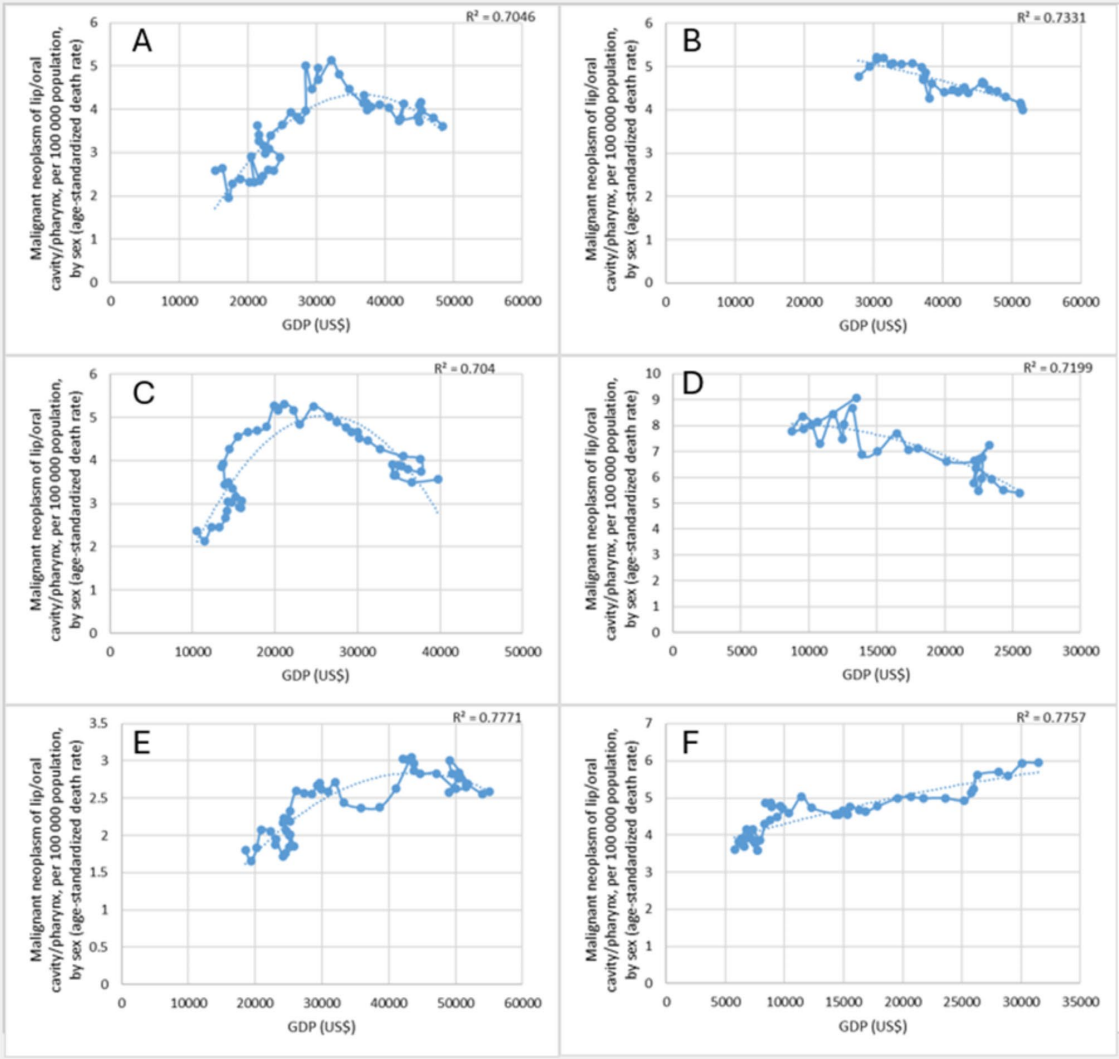
Relationship between the mortality rate of oral cancer and economic development **(A)** in Belgium, **(B)** in Germany, **(C)** in Spain, **(D)** in Croatia, **(E)** in the Netherlands and **(F)** in Poland

For 6 countries, the R^2^ values were equal or higher than 0.7, but lower than 0.8, namely, for Belgium, Germany, Spain, Croatia, The Netherlands and Poland (Fig. 1).

We analysed data from Belgium between 1968 and 2016, covering 49 years. We found that the age-specific mortality rate in 1968 was 2.58 per 100 000 inhabitants, rising to 3.61 in 2016. In 1997, the mortality rate reached its highest value, 5.13 per 100,000 inhabitants. The value of GDP in that year was US$ 32,135. In the previous years, the increase in mortality rate followed the growth rate of GDP, starting to decline in 1997. The coefficient of determination when fitting the second-degree curve was 0.705, indicating a good fit for the model. We analysed the data from 1990 to 2019 in Germany. In 1990, the GDP was US$ 27,885, in 2019 it was US$ 51,592. The age-standardised mortality rate was initially 4.77 per 100,000 inhabitants, in 2019 it was 3.99. In Germany, the mortality rate peaked in 1993. The death rate was 5.21 deaths per 100,000 inhabitants, with a GDP value of US$ 30,441. In the 26 years thereafter, the death rate has decreased in line with the increase of GDP. The coefficient of determination for the second-degree curve was equal to 0.733. According to data for Spain between 1968 and 2017, GDP was US$ 10,541 and US$ 39,730 respectively. The age-standardised mortality rate was 2.37 per 100,000 inhabitants in 1968 and 3.56 in 2017. The maximum mortality rate in Spain was 5.31 per 100,000 inhabitants, measured in 1994. The value of GDP in that year was US$ 21,152. Before 1994, the mortality rate increased, then decreased in line with the increase of GDP. The coefficient of determination was 0.704. Croatian data from 1990 to 2017 were examined, with a GDP of US$ 13,493 in 1990 and US$ 25,502 in 2017. The highest value of the age-standardised mortality rate in 1990 was 9.07per 100,000 inhabitants, which decreased to 5.4 in 2017. The coefficient of determination was 0.720.

We examined the data between 1969 and 2018 in the Netherlands. In 1969, the GDP value was US$ 18,585, in 2018 US$ 55,094. The age-adjusted death rate was 1.8 per 100,000 inhabitants in 1969, in 2018 it was 2.59. The Netherlands has the lowest maximum death rate among the examined countries − 3.05 per 100,000 inhabitants in 2000. In that year, the GDP was US$ 43,442.3. In previous years, the rate increased slightly in line with GDP growth, and then decreased from 2000 onwards. The coefficient of determination was 0.777. When examining the data for Poland between 1970 and 2018, the value of GDP was initially US$ 5,776, in 2018 it was US$ 31,497. The age-adjusted death rate was 3.61 per 100,000 inhabitants in 1970, which increased continuously in line with the growth of GDP, reaching 5.95 per 100,000 inhabitants in 2018. The coefficient of determination is 0.777.

**Fig. 2:**
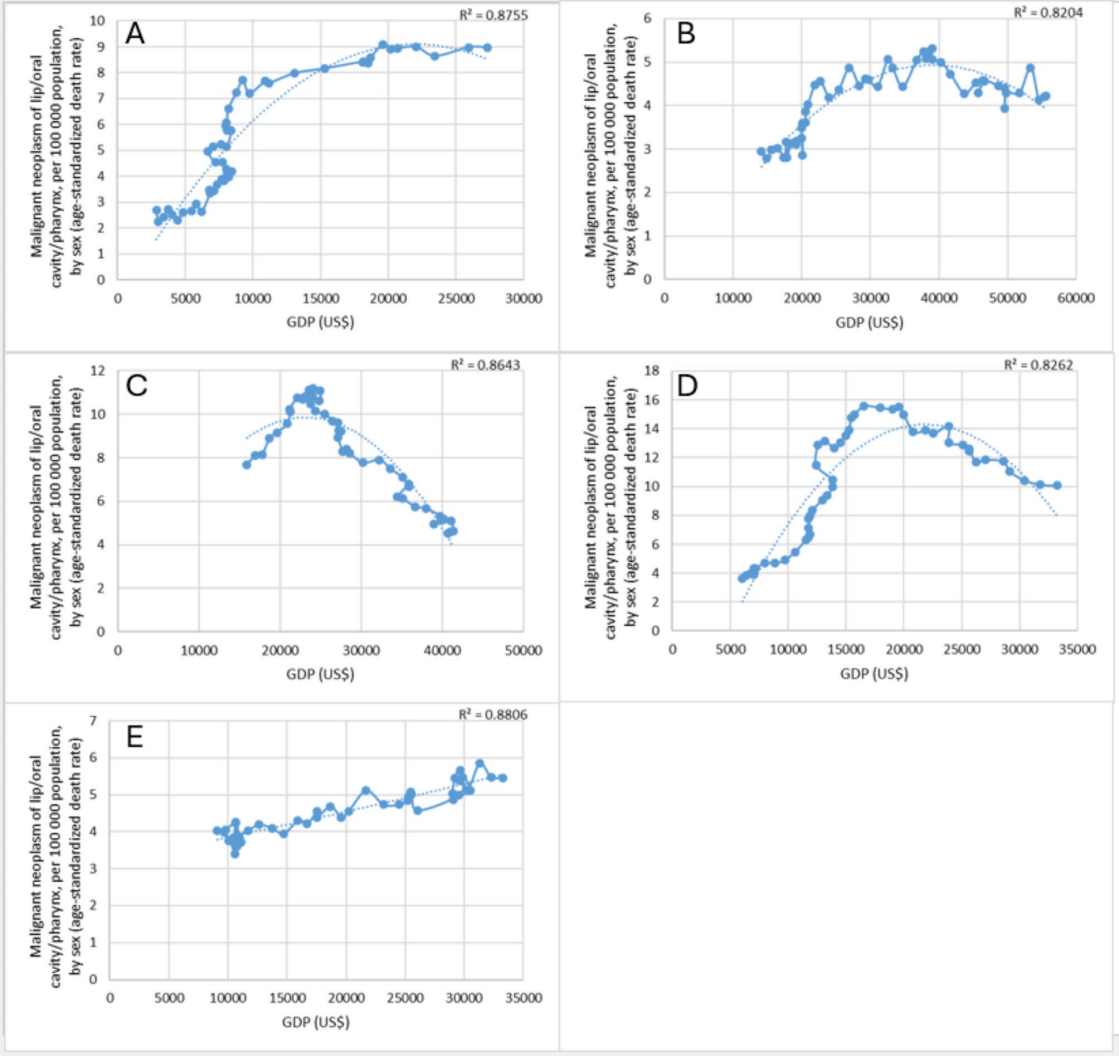
Relationship between the mortality rate of oral cancer and economic development **(A)** in Romania, **(B)** in Austria, **(C)** in France, **(D)** in Hungary and **(E)** in Portugal

For 5 countries, the R^2^ values were equal or above 0.8, but under 0.9, specifically, for Romania, Austria, France, Hungary and Portugal (Fig. 2). We examined the data between 1969 and 2018 in Romania. In 1969, the GDP was US$ 2,878, in 2018 US$ 27,323. The age-adjusted death rate was 2.69 per 100,000 inhabitants in 1969, and 8.97 in 2018. The death rate peaked at 9.09 deaths per 100,000 inhabitants in 2012, with a GDP value of US$ 19,606 in that year. In the previous years, the death rate increased in line with GDP growth, but decreased slightly for 6 years after 2012. The coefficient of determination is 0.876. For Austria, the data covered 51 years. In 1969, the GDP was US$ 14,091, in 2019 US$ 55,613. The age-standardised death rate per 100,000 inhabitants was 2.95 in 1969, it increased to 4.23 in 2019. The mortality rate peaked at 5.31 per 100,000 inhabitants in 2000, with a GDP value of US$ 38,984 that year. In the years before, the mortality rate increased in line with GDP growth. The coefficient of determination is 0.820 which implies a relatively strong correlation. The French data were analysed between 1968 and 2014. In 1968, the GDP was US$ 15,915, in 2014 it was US$ 40,729. The age-standardised mortality rate was initially 7.68 per 100,000 inhabitants, decreasing to 4.53 in 2014. The highest mortality rate was measured in 1981, 11.21 per 100,000 inhabitants. In that year, the GDP was US$ 24,074. Between 1968 and 1981, the death rate increased in line with GDP growth, then decreased. The coefficient of determination is 0.864. Looking at data from 1970 to 2019 in Hungary, GDP was US$ 6,053 in 1970 and US$ 33,265 in 2019. The age-adjusted mortality rate was 3.65 per 100,000 inhabitants in 1970, rising to 10.06 in 2019. The highest death rate in Hungary was 15.59 per 100,000 inhabitants in 2000. In that year, GDP was US$ 16,567. In the previous year’s mortality rate increased in line with GDP growth and then decreased. The R-squared value was 0.826. The Portuguese data were analysed between 1971 and 2018. The value of GDP was US$ 9,093 in 1971 and US$ 33,298 in 2018. The age-adjusted death rate was 4.02 per 100,000 inhabitants in 1971 and 5.46 in 2018. The highest death rate was recorded in 2016, 5.84 per 100,000 inhabitants, with a GDP of US$ 31,303. In the following two years, the death rate decreased slightly. The coefficient of determination is 0.881.

**Fig. 3:**
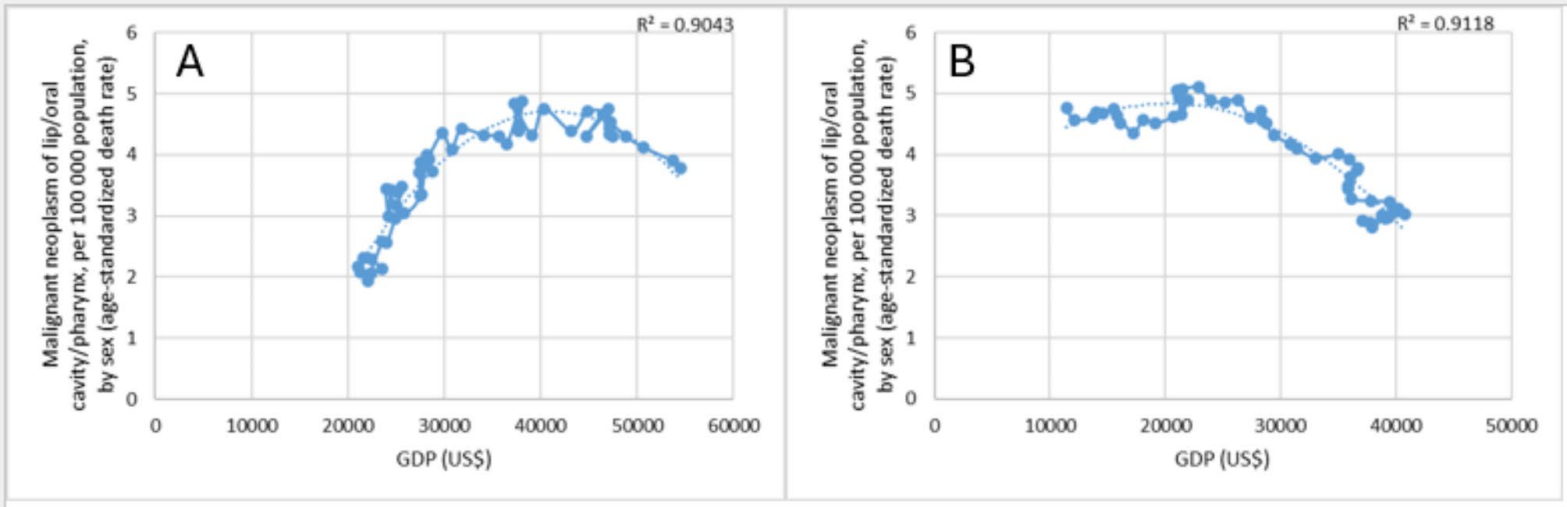
Relationship between the mortality rate of oral cancer and economic development **(A)** in Denmark and **(B)** in Italy

For Denmark and Italy the R^2^ values were equal or higher than 0.9 (Fig. 3). The Danish data were examined from 1969 to 2018. In 1969, the GDP was US$ 21,000, in 2018 it was US$ 54,500. The age-standardised death rate was 2.17 per 100,000 inhabitants in 1969, in 2018 it was 3.79. The highest death rate was 4.84 per 100,000 inhabitants in 2000. The value of GDP in that year was US$ 38,023. In the previous years, the death rate increased in line with GDP growth, reaching a maximum and then declining in line with GDP growth. The coefficient of determination is 0.904 which implies a very strong correlation. We analysed the Italian data between 1968 and 2017. In 1968 the GDP was US$ 11,462, in 2017 it was US$ 40,779. The age-adjusted mortality rate was 4.77 per 100,000 inhabitants in 1968, in 2017 decreased to 3.02. The highest mortality rate was 5.1 per 100,000 inhabitants, in 1986. In that year was US$ 22,860. In earlier years, the death rate increased slightly in relation to the increase in GDP, and then decreased from 1986. The coefficient of determination is 0.912.

Overall, in 10 out of the examined 13 countries mortality rate of oral cancer increased with GDP and then started to decrease above a certain income level.

### Spearman correlation and vector autoregression regarding GDP and oral cancer mortality rates

Spearman correlation analysis showed a strong positive association between GDP and oral cancer mortality in Belgium, with a correlation coefficient of 0.698, which was statistically significant (*p* < 0.001), suggesting that higher GDP levels were associated with higher oral cancer mortality rates (Table 1). Further analysis using a Vector Autoregressive (VAR) model revealed a non-significant relationship between changes in GDP and changes in oral cancer mortality within the analysed lags. Specifically, the coefficient for the first lag (L1) was − 2.92 × 10^-6 (*p* = 0.606) and for the second lag (L2) was − 1.32 × 10^-6 (*p* = 0.812), indicating no significant immediate impact of GDP changes on oral cancer mortality rates.

The Spearman correlation analysis indicated a relatively strong negative association between Germany’s GDP and oral cancer mortality rates, with the correlation coefficient equal to -0.863 with a p-value lower than 0.001, suggesting that higher GDP levels might be associated with lower oral cancer mortality rates in Germany. Changes in GDP did not have a significant immediate impact on changes in oral cancer mortality rates within the analysed lags, because the coefficient for L1 was 3.86 × 10^-7 (*p* = 0.253) and for L2 was − 6.68 × 10^-8 (*p* = 0.846). The Spearman correlation coefficient between Spain’s oral cancer mortality rates and GDP was 0.443, indicating a moderate positive association, which might suggest that higher GDP levels could be associated with higher oral cancer mortality rates in the country. The association was statistically significant with a p-value of 0.002, meaning there is strong evidence to reject the null hypothesis of independence between GDP and oral cancer mortality rates. The coefficient for the first lag of GDP is -1.67 × 10^-6, with a p-value of 0.082. Although the effect size could be considered as very weak, it is close to being statistically significant at the 10% level, suggesting a potential weak inverse relationship where an increase in GDP might slightly reduce the rate of increase in oral cancer mortality in the following year. The coefficient for the second lag of GDP is 3.20 × 10^-7, with a p-value of 0.750, but this effect was not statistically significant. In Croatia the correlation coefficient derived from the Spearman correlation was − 0.788 with a p-value lower than 0.001, which indicates that a negative association could be observed regarding oral cancer mortality and GDP. However, the VAR analysis indicates that changes in GDP (L1 = 1.46 × 10^-5, *p* = 0.660; L2 = 2.09 × 10^-5, *p* = 0.416) had a very minimal and statistically insignificant effect on the changes in oral cancer mortality rates in Croatia. The Spearman correlation between oral cancer mortality and GDP in the Netherlands was 0.793 (*p* < 0.001), which might indicate a strong and statistically significant positive relationship. The VAR model showed that changes in GDP (L1 = 6.51 × 10^-7, *p* = 0.594; L2 = 1.18 × 10^-6, *p* = 0.342) have no significant effect on changes in oral cancer mortality rates. The Spearman correlation coefficient regarding oral cancer mortality and GDP was 0.897 with a p-value lower than 0.001, indicating that a very strong and statistically significant positive relationship could be observed, which suggests that higher GDP levels might be associated with higher oral cancer mortality rates in Poland. Based on the results obtained from the VAR model, it was found that changes in GDP have no statistically proven significant effect on changes in oral cancer mortality rates. Specifically, the coefficients for both the first lag (L1=-1.16 × 10^-6, *p* = 0.397) and the second (L2 = 1.52 × 10^-6, *p* = 0.276) lag of GDP could be considered as very weak and statistically not significant. The correlation coefficient from the Spearman correlation was equal to 0.935 with a p-value lower than 0.001, which might indicate a very strong association between oral cancer mortality and GDP in Romania. However, according to the results of the VAR model, it was found that changes in GDP (L1=-1.95 × 10^-6, *p* = 0.528; L2=-2.41 × 10^-6, *p* = 0.469) have no statistically proven effect on the changes in oral cancer mortality rates. The Spearman correlation between oral cancer mortality and GDP in Austria was equal to 0.590 (*p* < 0.001), which highlighted a moderate to strong and statistically significant positive association between the variables. The first-time lag regarding GDP had a negligible and statistically insignificant effect on changes in oral cancer mortality rates (L1=-4.96 × 10^-8, *p* = 0.995), which means that GDP changes from the previous year did not significantly impact the observed year’s change in oral cancer mortality rates in Austria. However, the second lag of GDP (L2) had a weak, but statistically significant negative effect on changes in oral cancer mortality rates (L2 = 1.58 × 10^-5, *p* = 0.038), hence an increase in GDP two years prior was associated with a slight decrease in the rate of change in oral cancer mortality rates. Based on a Spearman correlation coefficient of -0.755 (*p* < 0.001), there was a relatively strong inverse association between GDP and oral cancer mortality in France. The VAR model for France indicated that changes in GDP had no significant impact on changes in oral cancer mortality rates, since non-significant coefficients for both the first lag (L1 = 4.88 × 10^-7, *p* = 0.619) and the second lag (L2=-5.38 × 10^-7, *p* = 0.584) were observed. With a Spearman’s rho of 0.682 and a p-value less than 0.001, there was a strong and statistically significant positive correlation between GDP and oral cancer mortality rate in Hungary, which indicated that as GDP increases, oral cancer mortality rates also tended to increase. The VAR model results for Hungary indicated that changes in GDP from the second lag did not significantly affect the changes in oral cancer mortality rates (L2 = 9.73 × 10^-6, *p* = 0.528). However, an increase in GDP from the first lag was significantly associated with a reduction in the change of oral cancer mortality rates (L1 = 3.83 × 10^-5, *p* = 0.009). The Spearman correlation coefficient regarding GDP and oral cancer mortality in Portugal was 0.902 with a p-value of less than 0.001, which indicates a very strong and statistically significant positive association, suggesting that as GDP increases, oral cancer mortality rates also tend to increase in the country. The VAR model results for Portugal indicated that changes in GDP did not have any statistically significant impact on changes in oral cancer mortality rates. The first lag of GDP (L1 = 1.13 × 10^-5, *p* = 0.108) and the second lag (L2 = 6.04 × 10^-6, *p* = 0.403) showed small positive effects. However, neither of these effects is statistically significant at conventional levels. The Spearman correlation analysis between oral cancer mortality rates and GDP in Denmark indicated a strong positive association with a Spearman’s coefficient of 0.819 and p-value lower than 0.001, the data shows a high degree of correlation between the two variables analysed, suggesting that as GDP increases, oral cancer mortality rates also tended to increase. The results of the VAR model indicated that changes in GDP did not have a statistically significant impact on changes in oral cancer mortality rates in country. Both the first (L1=-6.85 × 10^-6, *p* = 0.356) and second lag (L2=-4.76 × 10^-6, *p* = 0.554) of GDP showed relatively weak negative coefficients, but neither was significant at conventional levels. The correlation coefficient between oral cancer mortality and GDP in Italy was − 0.812 with a p-value lower than 0.001), which indicated a statistically significant and strong negative relationship. The VAR model results for Italy indicated that changes in GDP had a significant impact on changes in oral cancer mortality rates, with the first lag of GDP showing a small, non-significant positive effect (L1 = 7.75 × 10^-7, *p* = 0.091) and the second lag of GDP showing a small, statistically significant negative effect (L2=-1.12 × 10^-6, *p* = 0.017), suggesting that while recent GDP changes did not have a statistically proven immediate impact, GDP changes from two periods might be associated with a reduction in the current change of oral cancer mortality rates (Table 1).

**Table 1:** Summary of Spearman correlation coefficients and vector autoregression (VAR) model results for GDP and oral cancer mortality rates across 13 countries

Country	Spearman correlation		Vector autoregression (VAR)			
			Lag 1 (L1)		Lag 2 (L2)	
	Coefficient	*p*-value	Coefficient	*p*-value	Coefficient	*p*-value
**Austria**	0.590	< 0.001*	-4.96 × 10^-8	0.995	1.58 × 10^-5	0.038*
**Belgium**	0.698	< 0.001*	2.92 × 10^-6	0.606	-1.32 × 10^-6	0.812
**Germany**	-0.863	< 0.001*	3.86 × 10^-7	0.253	-6.68 × 10^-8	0.846
**Denmark**	0.819	< 0.001*	-6.85 × 10^-6	0.356	-4.76 × 10^-6	0.554
**Spain**	0.443	0.002*	-1.67 × 10^-6	0.082	3.20 × 10^-7	0.750
**France**	-0.755	< 0.001*	4.88 × 10^-7	0.619	-5.38 × 10^-7	0.584
**Croatia**	-0.788	< 0.001*	1.46 × 10^-5	0.660	2.09 × 10^-5	0.416
**Hungary**	0.682	< 0.001*	3.83 × 10^-5	0.009*	9.73 × 10^-6	0.528
**Italy**	-0.812	< 0.001*	7.75 × 10^-7	0.091	-1.12 × 10^-6	0.017*
**Netherlands**	0.793	< 0.001*	6.51 × 10^-7	0.594	1.18 × 10^-6	0.342
**Poland**	0.897	< 0.001*	1.16 × 10^-6	0.397	1.52 × 10^-6	0.276
**Portugal**	0.902	< 0.001*	1.13 × 10^-5	0.108	6.04 × 10^-6	0.403
**Romania**	0.935	< 0.001*	-1.95 × 10^-6	0.528	-2.41 × 10^-6	0.469

*Significant finding (*p* < 0.05)

## Discussion

This study analysed the relationship between GDP and oral cancer mortality across 13 countries in an ecological design. The findings revealed that in 10 countries, oral cancer mortality increased alongside GDP, in several cases peaking at a certain income level before declining. Statistical analyses highlighted significant associations, however, the vector autoregression analysis generally showed that changes in GDP had little immediate impact on shifts in oral cancer mortality, although some countries, like Hungary, Austria and Italy, exhibited minor delayed effects in later periods. However, short-term fluctuations in GDP do not appear to have an immediate impact on cancer mortality rates in the vast majority of countries. This might suggest that while GDP and mortality could be correlated, the timing and nature of their interaction can vary greatly. Furthermore, as a result of our descriptive analysis, we found that there is a relationship between the mortality rate of oral cancer and the economic development of a country. In the majority of the countries studied, there is a downward trend in oral cancer mortality rates as GDP increases.

Simon Kuznets, in an article entitled “Economic growth and income inequality”, examined the relationship between national income per capita and income inequality. He concluded that, in a given society, as income rises in the early stages of economic growth, income inequality rises in parallel. However, when per capita income reaches a certain level, i.e. when an income turning point occurs, income inequality starts to decline [23, 24]. The number of studies examining the health significance of the Kuznets curve is scarce and the available research has so far mainly analysed the association between economic well-being and obesity [25–27].

In 10 out of the examined 13 countries (Austria, Belgium, Germany, Denmark, Spain, France, Portugal, Hungary, Italy, the Netherlands, Croatia, Italy, Romania) mortality rate of oral cancer increased with GDP and then started to decrease above a certain income level. While in Portugal and Romania, the mortality rate fell slightly at the end of the examined period, the interval examined after the turning point was 2 years in case of Portugal and 6 years in case of Romania, so assessing the trend requires further investigation. However, in Poland the age-adjusted mortality rate of oral cancer increased steadily in line with GDP growth during the examined period and did not decrease until the end of the examined period (2018). Based on our analysis, 10 countries met the “traditional” Kuznets hypothesis, while 3 did not. Thus, for the exceptions, there was no turning point in a country’s economic development in a given period where the mortality rate of oral cancer stagnated or started to decline.

At the population level, oral cancer mortality can be influenced by the characteristics of the health care system, alcohol consumption, smoking, and the prevalence of HPV [28].

As studies have been written about the connection between alcohol consumption, smoking and economic status [29, 30], in countries not following the trend (such as Portugal, Romania, Poland), we looked at the financing of oral health care to identify the causes. As oral health is a special form of health care, where there is a close collaboration between dentists, general practitioners, dental and oral care specialists, we have to consider the financing of both the general health care and specifically dental care.

In the early 1980s, the public health system in Portugal faced a significant shortage of resources, which led to a high level of involvement of private providers through contracts and agreements. Access to dental care puts a significant financial burden on the Portuguese population, as they often have to pay the full cost of treatment. Since 2008, as part of a national programme to improve oral health, the public health system has been offering so-called “dentist vouchers”, which gave free dental care to women undergoing antenatal care, the elderly, children up to the age of 16, and patients infected with HIV/AIDS, with which they could receive free care from dentists under contract with the care system. In March 2014, in view of the high mortality and morbidity of oral cancer, the programme was extended to screening the main risk group. Accordingly, men over 40 years of age who smoke, regularly consume alcohol or have oral lesions identified by an oral surgeon or dentist are provided with a voucher every 2 years for screening and, if necessary, for biopsy [31].

In eastern Europe - the Czech Republic, Estonia, Hungary, Latvia, Lithuania, Poland, Slovakia and Slovenia - publicly funded oral health care had shifted to private care in the past, following political changes starting in 1989. The extent of this varies from country to country. Several countries are already planning to introduce insurance-based care [28].

In Poland, an additional problem is that only 15% of the adult population used the remaining publicly funded oral health services in 2014, which can be explained by the poor health awareness of the Polish society and the under-representation of oral care and oral health in Polish health policy. The level of public spending on oral health is relatively low and dentists providing publicly funded dental services mainly perform restorative treatment, with only a small number of preventive procedures due to underfunding [32].

In Romania, after the change of regime, the lack of state resources shifted dental care from being entirely state-funded to the private sector, with publicly funded dental care now only available at universities and the armed forces. In recent years, Romania has had the lowest oral health expenditure per capita in the European Union. Both the number of dentists under contract with the National Health Insurance House and the amount of state support have fallen dramatically, and between April 2013 and June 2014, no financial support was received by the public sector. During this period, the number of estimated dental visits decreased by a tenth, partly due to underfunding and partly due to underreporting by the private sector. According to 2007 data provided by the National Public Health Institute-National Centre for Statistics and Informatics in Public Health from the Ministry of Public Health, only 0.6% of the public budget for oral health care was spent on preventive activities [33].

Compared to the descriptive and Spearman correlation analysis, only a very few countries showed statistically significant lagged effects. Austria had a weak but significant positive effect with the second lag of GDP, Hungary had a significant negative effect with the first lag of GDP, and Italy had a significant negative effect with the second lag of GDP. In most cases, both the first and second lags of GDP changes were not statistically significant, indicating that short-term fluctuations in GDP do not directly influence changes in oral cancer mortality rates. Overall, while there are clear long-term associations between GDP and oral cancer mortality rates, the immediate causal effects of GDP changes on mortality rates are limited, suggesting that other factors and longer-term dynamics could play a more crucial role in this relationship. Further research is needed in order to explore these mediating factors and the complex interplay between economic conditions and health outcomes.

## Strengths and limitations

This study could be considered unique due to its topic. The possible relationship between the variables of interest could be observed based on univariate analyses; however, the conclusions drawn should not be interpreted on individual level data in order to avoid ecological fallacy. Although, the analyses revealed valuable associations, it should be noted that all analyses were univariate and did not include multiple analyses adjusted for potential confounders. Nevertheless, it is important to note that a clear causal link cannot be established because of the many other factors that could influence the results. The Health for All explorer uses interactive online tools to visualise regional and national differences of health indicators, so all data, graphs, charts and maps could be exported and are available free of charge for publication. The primary strength of the study is that around 50 years of data could be examined for several factors.

## Conclusion

The findings suggest that higher GDP levels are associated with higher oral cancer mortality rates in most countries, while a few countries exhibited the opposite trend. However, the VAR models indicated that changes in GDP generally did not have significant immediate impacts on changes in oral cancer mortality rates, highlighting the complexity of the relationship and suggesting that the effects of GDP on mortality may involve longer-term dynamics and other mediating factors. Specifically, while long-term associations were found, immediate economic changes did not directly influence mortality rates, highlighting the public health need for further investigation into the temporal and causal relationships between economic conditions and health outcomes.

Taking these into consideration, it can be said that an increase in a country’s economic development alone does not guarantee a decrease in the number of oral cancer patients. In order to reduce the number of cases of oral cancer, education and prevention are essential, as well as adequate financing of health care, including dental care, and the provision of optimally functioning primary dental care, accessible to the population free of charge.

## Data Availability

The data presented in this study are available to the public (World Health Organization, European Health Information Gateway: https://gateway.euro.who.int/en/hfa-explorer/ and Groningen Growth and Development Centre: https://www.rug.nl/ggdc/productivity/pwt/?lang=en).
